# Supplementary material for: Targeting fatty acid synthase reduces aortic atherosclerosis and inflammation
Source: Commun Biol. 2025 Feb 19;8:262. doi: 10.1038/s42003-025-07656-1 (PMC11840040; doi:10.1038/s42003-025-07656-1)
Supplement: Supplementary file 1 — Supplementary Information [file 42003_2025_7656_MOESM1_ESM.pdf]

Supplemental Figure 1

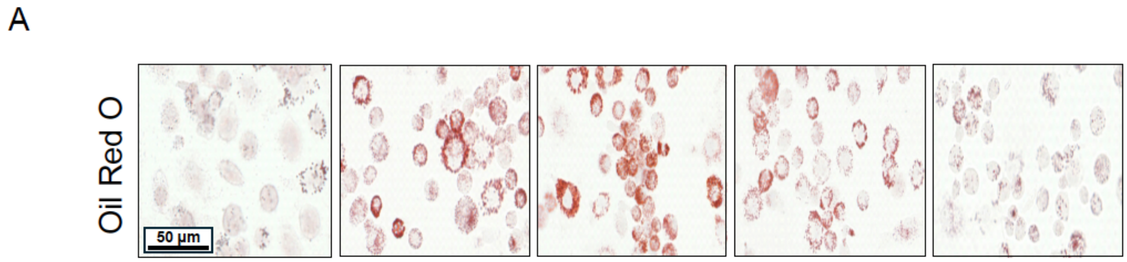

Healthy Serum Donor (%)	0	100	0	50	75
High cFAS/low LDL (%)	0	0	100	50	25

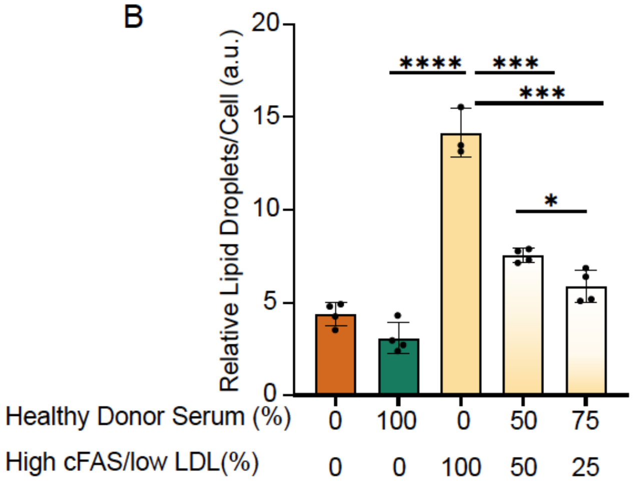

Effect of High cFAS/Low LDL Serum on Lipid Droplet Accumulation in Cells.

(A) Representative images of lipid droplets in macrophages treated with different serum conditions as stained by Oil Red O. The images display lipid accumulation in response to varying percentages of healthy donor serum versus high cFAS/low LDL serum. Conditions shown include 100% healthy donor serum, 100% high cFAS/low LDL serum, and mixtures of 50% healthy donor serum with 50% high cFAS/low LDL serum, as well as 75% healthy donor serum with 25% high cFAS/low LDL serum. Scale bar represents 50 µm.

(B) Quantification of lipid droplets per cell under each serum condition. Relative lipid

droplet accumulation per cell is significantly higher in cells treated with 100% high cFAS/low LDL serum compared to 100% healthy donor serum. Mixed conditions (50% healthy donor serum and 50% high cFAS/low LDL serum, as well as 75% healthy donor serum and 25% high cFAS/low LDL serum) show intermediate levels of lipid droplet accumulation. Data are mean ± SEM. ****$p < 0.0001$, ***$p < 0.001$, *$p < 0.05$.
