# Supplementary material for: Targeting fatty acid synthase reduces aortic atherosclerosis and inflammation
Source: Commun Biol. 2025 Feb 19;8:262. doi: 10.1038/s42003-025-07656-1 (PMC11840040; doi:10.1038/s42003-025-07656-1)
Supplement: Supplementary file 2 — Description of Additional Supplementary Files [file 42003_2025_7656_MOESM2_ESM.pdf]

Description of Additional Supplementary Files

File name: Supplementary Data 1

Description: Raw data of experiments and studies reported in the manuscript.
