# Supplementary material for: Targeting fatty acid synthase reduces aortic atherosclerosis and inflammation
Source: Commun Biol. 2025 Feb 19;8:262. doi: 10.1038/s42003-025-07656-1 (PMC11840040; doi:10.1038/s42003-025-07656-1)
Supplement: Supplementary file 4 — Reporting Summary [file 42003_2025_7656_MOESM4_ESM.pdf]

nature portfolio

Corresponding author(s): Mohamed Zayed, MD, PhD

Last updated by author(s): Jan 26, 2025

Reporting Summary

Nature Portfolio wishes to improve the reproducibility of the work that we publish. This form provides structure for consistency and transparency in reporting. For further information on Nature Portfolio policies, see our Editorial Policies and the Editorial Policy Checklist.

Statistics

For all statistical analyses, confirm that the following items are present in the figure legend, table legend, main text, or Methods section.

n/a	Confirmed	
☐	☒	The exact sample size (*n*) for each experimental group/condition, given as a discrete number and unit of measurement
☐	☒	A statement on whether measurements were taken from distinct samples or whether the same sample was measured repeatedly
☐	☒	The statistical test(s) used AND whether they are one- or two-sided<br>*Only common tests should be described solely by name; describe more complex techniques in the Methods section.*
☐	☒	A description of all covariates tested
☐	☒	A description of any assumptions or corrections, such as tests of normality and adjustment for multiple comparisons
☐	☒	A full description of the statistical parameters including central tendency (e.g. means) or other basic estimates (e.g. regression coefficient) AND variation (e.g. standard deviation) or associated estimates of uncertainty (e.g. confidence intervals)
☐	☒	For null hypothesis testing, the test statistic (e.g. *F*, *t*, *r*) with confidence intervals, effect sizes, degrees of freedom and *P* value noted<br>*Give P values as exact values whenever suitable.*
☒	☐	For Bayesian analysis, information on the choice of priors and Markov chain Monte Carlo settings
☒	☐	For hierarchical and complex designs, identification of the appropriate level for tests and full reporting of outcomes
☒	☐	Estimates of effect sizes (e.g. Cohen's *d*, Pearson's *r*), indicating how they were calculated

Our web collection on statistics for biologists contains articles on many of the points above.

Software and code

Policy information about availability of computer code

Data collection	N/A
Data analysis	N/A

For manuscripts utilizing custom algorithms or software that are central to the research but not yet described in published literature, software must be made available to editors and reviewers. We strongly encourage code deposition in a community repository (e.g. GitHub). See the Nature Portfolio guidelines for submitting code & software for further information.

Data

Policy information about availability of data

All manuscripts must include a data availability statement. This statement should provide the following information, where applicable:

- Accession codes, unique identifiers, or web links for publicly available datasets
- A description of any restrictions on data availability
- For clinical datasets or third party data, please ensure that the statement adheres to our policy

All data will be made available to reasonable requests placed to the corresponding author.

Research involving human participants, their data, or biological material

Policy information about studies with human participants or human data. See also policy information about sex, gender (identity/presentation), and sexual orientation and race, ethnicity and racism.

Reporting on sex and gender	We report that all serum samples used were from male individuals with similar age demographics.
Reporting on race, ethnicity, or other socially relevant groupings	N/A
Population characteristics	N/A
Recruitment	We report that serum samples were obtained from the Washington University in St. Louis vascular biobank.
Ethics oversight	We report that the vascular biobank is IRB approved.

Note that full information on the approval of the study protocol must also be provided in the manuscript.

Field-specific reporting

Please select the one below that is the best fit for your research. If you are not sure, read the appropriate sections before making your selection.

☒ Life sciences ☐ Behavioural & social sciences ☐ Ecological, evolutionary & environmental sciences

For a reference copy of the document with all sections, see nature.com/documents/nr-reporting-summary-flat.pdf

Life sciences study design

All studies must disclose on these points even when the disclosure is negative.

Sample size	No sample size estimations were performed. We used generally accepted methods to perform in vitro cell experiments at least n=3, and murine animal assessments using at least n=6.
Data exclusions	No data was excluded
Replication	All data were replicated at least with n=3 independent data repeats. Raw data file is provided in Supplemental Data 1.
Randomization	N/A
Blinding	When applicable human and mouse groups were blinded to the experiment operator.

Reporting for specific materials, systems and methods

We require information from authors about some types of materials, experimental systems and methods used in many studies. Here, indicate whether each material, system or method listed is relevant to your study. If you are not sure if a list item applies to your research, read the appropriate section before selecting a response.

Materials & experimental systems

n/a	Involved in the study	
☐	☒	Antibodies
☐	☒	Eukaryotic cell lines
☒	☐	Palaeontology and archaeology
☐	☒	Animals and other organisms
☒	☐	Clinical data
☒	☐	Dual use research of concern
☒	☐	Plants

Methods

n/a	Involved in the study	
☒	☐	ChIP-seq
☒	☐	Flow cytometry
☒	☐	MRI-based neuroimaging

Antibodies

Antibodies used	FAS antibody (Santa Cruz, SC-48357), anti-CD68 antibody (Bio Rad, MCA1957), antibody donkey anti-rat IgG labeled with Alexa Fluor® 555 (Thermo Fisher Scientific, A78945)

Validation	1. FAS Antibody (Santa Cruz, SC-48357): According to the manufacturer's datasheet, this antibody is validated for use for immunohistochemistry and has been tested for reactivity in human cells. Relevant peer-reviewed literature has reported its successful use in human tissue. Antibody specificity was further confirmed through the inclusion of appropriate positive and negative controls within our experimental setup using control and experimental macrophages.<br>2. Anti-CD68 Antibody (Bio-Rad, MCA1957): Manufacturer documentation indicates validation for immunohistochemistry and reactivity with human and murine species. Previous studies have utilized this antibody for detecting CD68 expression in human and murine endothelial cells. In this study, antibody performance was verified through staining patterns consistent with known CD68 staining relative to H&E staining<br>3. Donkey Anti-Rat IgG Alexa Fluor® 555 (Thermo Fisher Scientific, A78945): The secondary antibody was validated by the manufacturer for fluorescence-based applications such as immunofluorescence and flow cytometry in rat samples. Specificity was ensured through no-primary-antibody controls to rule out non-specific binding. The antibody is included in established online antibody databases such as Thermo Fisher's product profile, supporting its reliability in detecting rat IgG with minimal cross-reactivity.

Eukaryotic cell lines

Policy information about cell lines and Sex and Gender in Research

Cell line source(s)	Human U-937 cells (ATCC # CRL-1593.2)
Authentication	1. Supplier Validation (ATCC): The U-937 cell line was obtained from the American Type Culture Collection (ATCC), which provides authentication through short tandem repeat (STR) profiling to confirm cell identity. According to ATCC documentation, U-937 cells are validated for use in immunology, cell differentiation studies, and apoptosis research, with recommended culture conditions provided to maintain phenotype and functionality.<br>2. Published Literature: The U-937 cell line has been extensively characterized in peer-reviewed studies for its ability to differentiate into macrophage-like cells in response to stimuli such as phorbol 12-myristate 13-acetate (PMA). Literature confirms their suitability for studying monocytic function, inflammatory responses, and immune signaling pathways.
Mycoplasma contamination	Prior to experimental use, the cells were routinely tested for mycoplasma contamination using PCR-based detection methods to ensure culture integrity. Cell viability, morphology, and differentiation potential were assessed under phase-contrast microscopy and compared to documented characteristics. Functional assays, including response to differentiation-inducing agents, were performed to validate expected cell behavior.
Commonly misidentified lines<br>(See ICLAC register)	N/A

Animals and other research organisms

Policy information about studies involving animals; ARRIVE guidelines recommended for reporting animal research, and Sex and Gender in Research

Laboratory animals	C57BL/6 mice
Wild animals	N/A
Reporting on sex	We report the use of male mice.
Field-collected samples	Study did not involve samples collected from the field.
Ethics oversight	We report that all animal protocols were approved by the Washington University in St. Louis institutional animal care and use committee (IACUC). Mouse housing, breeding, and experimental procedures were conducted in accordance with national and institutional guidelines and ethics. All animals were humanely euthanized in accordance with the guidelines set forth by the IACUC and the American Veterinary Medical Association (AVMA) Guidelines for the Euthanasia of Animals

Note that full information on the approval of the study protocol must also be provided in the manuscript.

Plants

Seed stocks	N/A
Novel plant genotypes	N/A
Authentication	N/A
